# Prognostic implications of left ventricular hypertrophy defined by the thresholds from the international and Chinese guidelines

**DOI:** 10.1111/jch.14687

**Published:** 2023-06-19

**Authors:** Dan Zhou, Mengqi Yan, Anping Cai, Qiu Xie, Qi Cheng, Songtao Tang, Yingqing Feng

**Affiliations:** ^1^ Department of Cardiology Guangdong Cardiovascular Institute Guangdong Provincial People's Hospital Guangdong Academy of Medical Sciences Guangzhou People's Republic of China; ^2^ Department of Cardiology Guangdong Provincial People's Hospital (Guangdong Academy of Medical Sciences) Southern Medical University Guangzhou People's Republic of China; ^3^ Department of Internal Medicine Community Health Center of Liao Bu Community Dongguan People's Republic of China

**Keywords:** all‐cause mortality, cardiovascular mortality, Chinese threshold, guideline thresholds, left ventricular hypertrophy

## Abstract

To compare the predictive value of mortality between left ventricular hypertrophy (LVH) defined by Chinese thresholds and defined by international guidelines in hypertension individuals and investigate better indexation methods for LVH in Chinese population. We included 2454 community hypertensive patients with Left ventricular mass (LVM) and relative wall thickness. LVM was indexed to body surface area (BSA), height^2^ ^7^ and height ^1^ ^7^. The outcomes were all‐cause and cardiovascular mortality. Cox proportional hazards models were used to explore the association between LVH and the outcomes. C‐statistics and time‐dependent receiver operating characteristic curve (ROC) was used to evaluate the value of those indicators. During a median follow‐up of 49 months (interquartile range 2–54 months), 174 participants (7.1%) died from any cause (*n* = 174), with 71 died of cardiovascular disease. LVM/BSA defined by the Chinese thresholds was significantly associated with cardiovascular mortality (HR: 1.63; 95%CI: 1.00‐2.64). LVM/BSA was significantly associated with all‐cause mortality using Chinese thresholds (HR: 1.56; 95%CI: 1.14‐2.14) and using Guideline thresholds (HR: 1.52; 95%CI: 1.08‐2.15). LVM/Height^1.7^ was significantly associated with all‐cause mortality using Chinese thresholds (HR: 1.60; 95%CI: 1.17‐2.20) and using Guideline thresholds (HR: 1.54; 95%CI: 1.04‐2.27). LVM/Height^2.7^ was not significantly associated with all‐cause mortality. C‐statistics indicated that LVM/BSA and LVM/Height^1.7^ by Chinese thresholds had better predictive ability for mortality. Time‐ROC indicated that only LVM/Height^1.7^ defined by Chinese threshold had incremental value for predicting mortality. We found that in community hypertensive populations, race‐specific thresholds should be used to classify LV hypertrophy related to mortality risk stratification. LVM/BSA and LVM/Height^1.7^ are acceptable normalization method in Chinese hypertension.

## 1 INTRODUCTION

Hypertension was an important risk factor for cardiac morbidity and mortality.^1^ Left ventricular hypertrophy (LVH), mainly pathologically increased left ventricular mass, independent contributed to cardiovascular events in diverse populations, including hypertension.^2^, ^3^, ^4^, ^5^, ^6^, ^7^ Over the last 3 decades, preventing LVH or pharmacological LV mass (LVM) reduction or reverse LVH could reduce cardiovascular events in hypertension has been demonstrated. Despite this success, there are a number of controversies surrounding the definition of LVH. WASE study^8^ has been demonstrated that the normal values of most 2D echocardiographic parameters differed significantly among different races and countries, across age, sex.

Although the American Society of Echocardiography (ASE) and the European Association of Cardiovascular Imaging (EACVI) guideline has recommended LVM and relative wall thickness (RWT) reference value, normal values of these echocardiographic parameters differ significantly among different racial and ethnic groups.^8^ The Echocardiographic Measurements in Normal Chinese Adults (EMINCA) study, performed in China, revealed that most echocardiographic normal values for the health Chinese were smaller than those recommended by the ASE/EACVI guideline.^9^ Recently, the same investigators found that using the ASE/EACVI guideline thresholds would overestimate the proportion of abnormal LV geometry in the Chinese hypertensive patients. They subsequently proposed the Chinese thresholds to define LVH and LV geometry patterns.^10^ These findings once again support the notion that ethnic‐specific normal reference values to defined LV geometric patterns are needed.

LVH is also determined by body size, apart from age, sex and race. One method for overcoming the limitation has been indexing LVM to body size. Most commonly this is done by dividing LVM by body surface area, height^1^ ^.^ ^7^ or height^2^ ^.^ ^7^. The prognostic value of LVH defined by different body size should be certified in large population‐based cohorts so that the best indexing methods can be ascertained in Chinese hypertension. Therefore, the aims of the current study were to (a) quantify and compare the prognostic value of LVH defined by the Chinese thresholds and the ASE/EACVI guideline and (b) the prognostic value of LVH indexed by different methods.

## 2 METHODS

### 2.1 Study population

**FIGURE 1:**
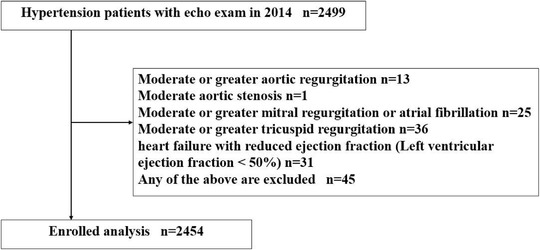
Study flow chart.

This was a community‐based cohort study conducted in Liao Bu County, Dongguan, Guangdong Province, China, as previously reported.^11^, ^12^ During the annual health examination sponsored by government in 2014, a total of 2499 hypertensive patients had echocardiographic examination. Hypertension was defined as systolic blood pressure (SBP) ≥140 mmHg or diastolic blood pressure (DBP) ≥90 mmHg and/or the use of antihypertensive medicine within 2 weeks. The inclusion criteria were hypertensive patients with sinus rhythm. The exclusion criteria were patients with history of myocardial infarction, significant valvular heart disease (moderate or greater valves regurgitation or stenosis), cardiomyopathy, heart failure with reduced ejection fraction (left ventricular ejection fraction <50%), atrial fibrillation, and with a poor imaging quality. Patients with established cancer were excluded. Finally, 2454 hypertensive patients were included in the current study (Figure 1). The study was performed in accordance with the principles of the Declaration of Helsinki and was approved by the Clinical Research Ethics Committee of Guangdong Provincial People's Hospital and the Liao Bu Community Health Center. Written informed consent was obtained before enrolment.

### 2.2 Clinical characteristics and laboratory examination

Clinical characteristics, including age, sex, prior medical history and current medication use, were collected by trained community healthcare staffs using standardized questionnaire. Weight and height were measured with participants standing without wearing heavy clothes or shoes by trained healthcare staffs. Body mass index (BMI) was calculated by weight in kilograms divided by height in squared meters. BMI ≥ 28 kg/m^2^ was defined as obesity.^13^ Waist circumference was measured at the level of the midpoint between the top of the iliac crest and the lower margin of the last palpable rib in the midaxillary line. Body surface area (BSA) was calculated as follow: BSA (m^2^) = (Weight [kg]^0.425^ * Height [cm]^0.725^) * 0.007184. Fasting venous blood was used to assess fasting plasma glucose (FPG), lipid profiles, and serum creatinine, which was used to calculate estimated glomerular filtration rate (eGFR) using the Modification of Diet in Renal Disease formula,^14^ and eGFR <60 mL/min/1.73 m^2^ was defined as chronic kidney disease (CKD).

### 2.3 Blood pressure measurement

Participants were required to sit in a quiet room for 5 min before blood pressure (BP) measurements. According to the Chinese hypertension guideline,^15^ the individuals kept the upper arm at the heart level and BP was measured using the Omron HEM‐7051 device (Omron HealthCare, Kyoto, Japan). Two BP measurements should be taken with 1 min interval and the average value was used. An additional measurement was required if the first two readings differ by >5 mmHg, and the mean value of the three readings was recorded.

### 2.4 Echocardiographic examination

Echocardiographic examination was performed by two experienced sonographers from Guangdong Provincial People's Hospital, using a Vivid S6 M4S‐RS Probe (GE Ving‐Med) interfaced with a 2.5‐ to 3.5‐MHz phased‐array probe. Left ventricular (LV) parameters were measured using standard 2D echocardiography according to the American Society of Echocardiography and the European Association of Cardiovascular Imaging (ASE/EACVI) guideline recommendation. Interventricular septal end‐diastolic thickness (IVSTd), LV posterior wall end‐diastolic thickness (LVPWTd), and LV end‐diastolic diameter (LVEDD) were recorded from the parasternal long‐axis view at the level of the mitral valve leaflet tips. LVM was calculated as follow: LVM = 0.8* 1.04 *[(IVSTd + LVEDD + LVPWTd)^3^ ‐ LVEDD^3^] + 0.6 g. LV ejection fraction was measured in the apical four‐ and two‐chamber views using the biplane Simpson method. Relative wall thickness (RWT) was calculated as: RWT = (LVPWTd*2)/LVEDD.

### 2.5 Definitions of LVH

The echocardiographic values of LVM were indexed to BSA and height^2^ ^.^ ^7^ based on the ASE/EACVI recommendations^16^, ^17^ and the European Society of Cardiology (ESC) and European Society of Hypertension (ESH) recommendations.^18^ We also used another indexation method by Chirinos and colleagues^19^ who proposed to index LVM to H^1.7^. Chinese thresholds were generated from recently published research.^10^ LVH thresholds were showed in Table S1.

### 2.6 Outcome

The outcome was all‐cause mortality and cardiovascular mortality. Causes of cardiovascular mortality included ischemic heart disease, heart failure, stroke. Others were all‐cause mortality. Outcomes were obtained from the dataset linkage to Dongguan Medical Insurance Bureau and Liao Bu Community Health Center. This captures the death registry and all admission data when submitting medical expense. Patients were censored at the time of outcome or at the end of follow‐up (December 31, 2018).

### 2.7 Interobserver variability

To test the reproducibility of echocardiographic measurements, three key parameters, including IVSTd, LVEDD and LVPWTd, were remeasured in 200 randomly selected subjects from the study participants. Interobserver variability was assessed between two investigators (Z.D. and X.Q.). Intraobserver variability was assessed by Z.D. Bland‐Altman analysis was used to assess interobserver and intraobserver variability and interclass correlation coefficients were calculated (Figure S1).

### 2.8 Statistical analysis

Continuous variables were presented as mean ± standard deviation if normal distribution, otherwise were presented as median and interquartile range (IQR). Categorical variables were presented as number (proportion). For categorical variables, the chi‐square test or Fisher exact test were used. When the continuous data were normal distribution, two groups were compared using the paired t‐test. Non‐parametric tests were used for analyzing data that were skew distribution. Univariable and multivariable Cox proportional hazards models were used to explore the association between LVH and outcome. Model 1 was unadjusted and model 2 was adjusted for age, sex, current smoking, waist circumference, BMI, SBP, heart rate, total cholesterol, eGFR, CKD, diabetes mellitus (T2DM), coronary artery disease (CAD), stroke, anti‐hypertension drugs with a forward stepwise procedure using the maximum likelihood ratio test. Hazard Ratio (HR) and 95% confidence interval (CI) were reported. Akaike Information Criterion (AIC) was provided to compare models. Kaplan−Meier curves were used to evaluate survival, and log‐rank tests were performed to analyze the differences. C‐statistics and time‐dependent receiver operating characteristic curves (time‐ROCs) were used to assess the predictive accuracy of each indicator. All the analyses were performed using IBM SPSS Statistics 25.0 software and R statistical software version 4.2.2. and a *p*‐value <.05 was considered as statistical significance.

## 3 RESULTS

### 3.1 Baseline characteristics

Among the 2454 hypertensive patients (mean age 63 years, 40.2% men) (Table 1), 57.9% had uncontrolled hypertension, 36.1% had mild hypertension, 16.8% had moderate hypertension, 5% had severe hypertension. In addition, 22.3% had DM, 20.2% CKD, 2.5% CAD, 3.6% stroke, and 26.4% obesity. 86% individuals were using antihypertensive drugs. The most commonly used drugs were angiotensin receptor blocker (52.7%) and calcium channel blockers (44.2%). The mean IVSTd, LVEDD and LVPWTd were 10.1 mm, 44.8 mm and 9.7 mm, respectively. The mean RWT and LVM/BSA were 0.43 and 93.9g/m^2^. The baseline characteristics comparison between participants with and without mortality were presented in Table 1.

**TABLE 1:** Baseline characteristics and echocardiographic measurements of hypertensive patients with or without mortality during follow‐up.

	Total *n* = 2454	With mortality (*n* = 174)	Without mortality (*n* = 2280)	*p*‐value
Age (years)	63 ± 11	75 ± 10	62 ± 11	<.001
Gender, Male (%)	986 (40.2%)	84 (48.3)	902 (39.6)	.024
Current smoking (%)	594 (24.2%)	65 (37.4%)	529 (23.2%)	<.001
Height (m)	1.57 ± 0.09	1.53 ± 0.10	1.57 ± 0.08	<.001
Weight (kg)	63.9 ± 12.3	58.53 ± 12.9	64.32 ± 12.19	<.001
Waist circumference (cm)	90.5 ± 9.9	90.2 ± 10.2	90.5 ± 9.9	.680
BMI (kg/m^2^)	25.8 ± 4.0	24.6 ± 4.4	25.9 ± 3.9	<.001
BSA (m^2^)	1.63 ± 0.18	1.55 ± 0.19	1.64 ± 0.18	<.001
SBP (mmHg)	143 ± 18	143 ± 21	143 ± 18	.678
DBP (mmHg)	81 ± 12	76 ± 13	81 ± 11	<.001
Heart rate (bpm/min)	74 ± 11	76 ± 12	74 ± 11	.025
FPG (mmol/L)	5.6 ± 1.6	5.7 ± 2.1	5.6 ± 1.5	.369
TC (mg/dL)	216 ± 48	207 ± 51	217 ± 47	.010
TG((mg/dL))	162 ± 149	154 ± 110	163 ± 149	.421
LDL cholesterol (mg/dL)	119 ± 34	114 ± 37	119 ± 33	.066
HDL cholesterol (mg/dL)	57 ± 14	55 ± 12	57 ± 14	.054
eGFR mL/min/1.73 m^2^	76 ± 20	59 ± 22	77 ± 19	<.001
T2DM (%)	547 (22.3)	52 (29.9)	495 (21.7)	.013
CKD (%)	496 (20.2)	85 (48.8)	411 (18.0)	<.001
CAD (%)	62 (2.5)	9 (5.2)	53 (2.3)	.021
Stroke (%)	89 (3.6)	16 (9.2)	73 (3.2)	<.001
Obesity (%)	649 (26.4)	38 (21.8)	611 (26.8)	.153
Anti‐hypertension drugs				
ACEI (%)	278 (11.3)	20 (11.5)	258 (11.3)	.943
ARB (%)	1294 (52.7)	82 (47.1)	1212 (53.2)	.125
Beta Blocker (%)	294 (12)	27 (15.5)	267 (11.7)	.136
Calcium Channel Blockers (%)	1085 (44.2)	78 (44.8)	1007 (44.2)	.866
Diuretic (%)	224 (9.1)	16 (9.2)	208 (9.1)	.974
IVSTd (mm)	10.1 ± 1.3	10.5 ± 1.4	10.1 ± 1.3	<.001
LVEDD (mm)	44.8 ± 3.6	44.7 ± 4.6	44.8 ± 3.5	.605
LVEDD/BSA (mm/m^2^)	27.6 ± 3.1	29.0 ± 3.9	27.5 ± 3.0	<.001
LVPWTd (mm)	9.7 ± 1.2	10.0 ± 1.3	9.6 ± 1.2	.001
RWT	0.43 ± 0.06	0.45 ± 0.07	0.43 ± 0.06	<.001
LVM (g)	153 ± 34	160 ± 39	152 ± 34	.004
LVM/BSA (g/m^2^)	93.9 ± 20.4	103.8 ± 26.0	93.2 ± 19.7	<.001
LVEF (%)	66 ± 5	65 ± 6	66 ± 5	.106

*Note*: Data are mean ± standard deviation or number (proportion)

Abbreviations: BMI, body mass index; BSA, body surface area; SBP, systolic blood pressure; DBP, diastolic blood pressure; IVSTd, interventricular septum thickness at end‐diastole; LVEDD, left ventricular end‐diastolic diameter; LVEF, left ventricular ejection fraction; LVM, left ventricular mass; LVPWTd, left ventricular posterior wall thickness at end‐diastole; RWT, relative wall thickness; FPG: fast plasma glucose; TC: total cholesterol; TG: Triglycerides; HDL‐C: high‐ density lipoprotein; LDL: low density Lipoprotein; eGFR: estimate glomerular filtration rate; ACEI: angiotensin converting enzyme inhibitors; ARB: Angiotensin Receptor Blocker; T2DM: type 2 diabetes mellitus; CKD: chronic kidney disease; CAD: coronary heart disease.

The total proportion of LVH when using the Chinese thresholds compared to the international guideline thresholds was presented in Figure S2 by index BSA, Height^1^ ^.^ ^7^ and Height^2^ ^.^ ^7^. When LVM was indexed to BSA or Height^1^ ^.^ ^7^, the prevalence of LVH showed significant higher using guidelines thresholds. The prevalence of LVH was significantly higher when indexed to Height^1^ ^.^ ^7^ using guidelines thresholds likely due to a much lower threshold from the Chirinos and colleagues^19^ than Chinese threshold in women. However, when LVM was indexed to Height^2^ ^.^ ^7^, the prevalence of LVH was similar between the two thresholds.

### 3.2 Outcomes

During a median follow‐up of 49 months (IQR 2–54 months), the all‐cause mortality rate was 7.1% (*n* = 174) and annual rate was 15.7 ‰, with cardiovascular mortality rate was 2.9% (*n* = 71) and annual rate was 6.4 ‰.

### 3.3 The association of LVH with cardiovascular mortality

**FIGURE 2:**
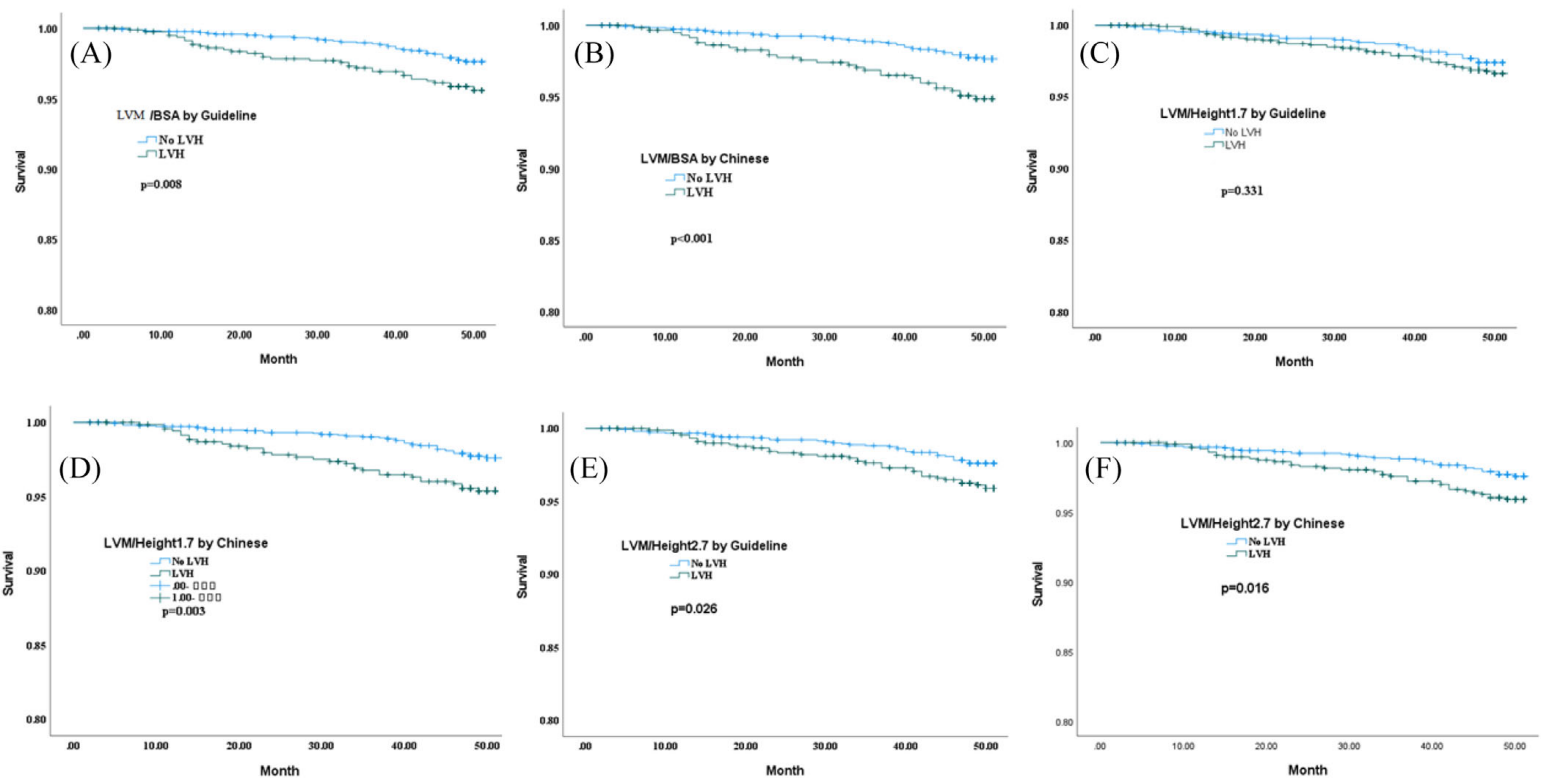
Kaplan‐Meier survival estimates of LVH and cardiovascular mortality by different thresholds, LVH, left ventricular hypertrophy. (A) LVH defined by LVM/BSA by Guideline. (B) LVH defined by LVM/BSA by Chinese. (C) LVH defined by LVM/Height^1.7^ by Guideline. (D) LVH defined by LVM/Height^1.7^ by Chinese. E: LVH defined by LVM/Height^1.7^ by Guideline. F: LVH defined by LVM/Height^1.7^ by Chinese.

Analysis was performed with indexing LVM to BSA, height ^1.7^and height^2^ ^.^ ^7^, respectively using different thresholds. Kaplan‐Meier curve showed that hypertensive patients with LVH, which using the LVM/BSA and LVM/Height^2.7^, defined by the international guideline and the Chinese thresholds had higher mortality risk (Figure 2A,B,E,F; Log‐rank ratio, all *p*<.05). LVH defined by LVM/Height^1.7^ using Chinese thresholds, not guideline threshold, had higher risk of mortality (Figure 2C,D). After adjusting for covariates, only LVH, which using the LVM/BSA, defined by the Chinese thresholds was associated with cardiovascular mortality (HR: **1.63**, 95%CI: 1.00‐2.64) (Table 2). LVH defined by LVM/BSA using Chinese threshold had the lowest AIC.

**TABLE 2:** Comparison of the effect of LVH defined by Chinese thresholds or Guideline thresholds on cardiovascular mortality.

	Model 1	Model 2		Model 1	Model 2	
	Chinese thresholds* HR (95% CI)	Chinese thresholds HR (95% CI)	AIC	Guideline thresholds HR (95% CI)	Guideline thresholds HR (95% CI)	AIC
LVH						
LVM/BSA^a^	2.33 (1.44‐3.75)	**1.63 (1.00‐2.64)**	1011.47	1.90 (1.19‐3.05)	1.66 (0.96‐2.85)	1012.71
LVM/Height^1.7^ ^b^	2.07 (1.29‐3.32)	1.50 (0.93‐2.43)	1012.95	1.39 (0.86‐2.25)	1.61 (0.85‐3.08)	1014.38
LVM/Height^2.7^ ^c^	1.82 (1.14‐2.91)	1.28 (0.75‐2.19)	1014.55	1.74 (1.09‐2.79)	1.25 (0.72‐2.18)	1014.60

*Note*: Model 1: unadjusted

Model 2: adjusted for age, sex, current smoking, waist circumference, body mass index, systolic blood pressure, heart rate, total cholesterol, estimated glomerular filtration rate, type 2 diabetes mellitus, chronic kidney disease, coronary heart disease, stroke, anti‐hypertension drugs.

LVM: left ventricular mass; LVH, left ventricular hypertrophy; HR, hazard ratio; AIC: Akaike Information Criterion.

* Thresholds defined left ventricular and atrial remodeling in hypertensive patients using thresholds from international guidelines and EMINCA data.^10^

^a^ Guideline threshold from recommendations for cardiac chamber quantification by echocardiography in adults: an update from the ASE and EACVI.^16^

^b^ Left ventricular mass: allometric scaling, normative values, effect of obesity, and prognostic performance.^19^

^c^ 2018 ESC/ESH Guidelines for the management of arterial hypertension.^18^

### 3.4 The association of LVH and all‐cause mortality

**FIGURE 3:**
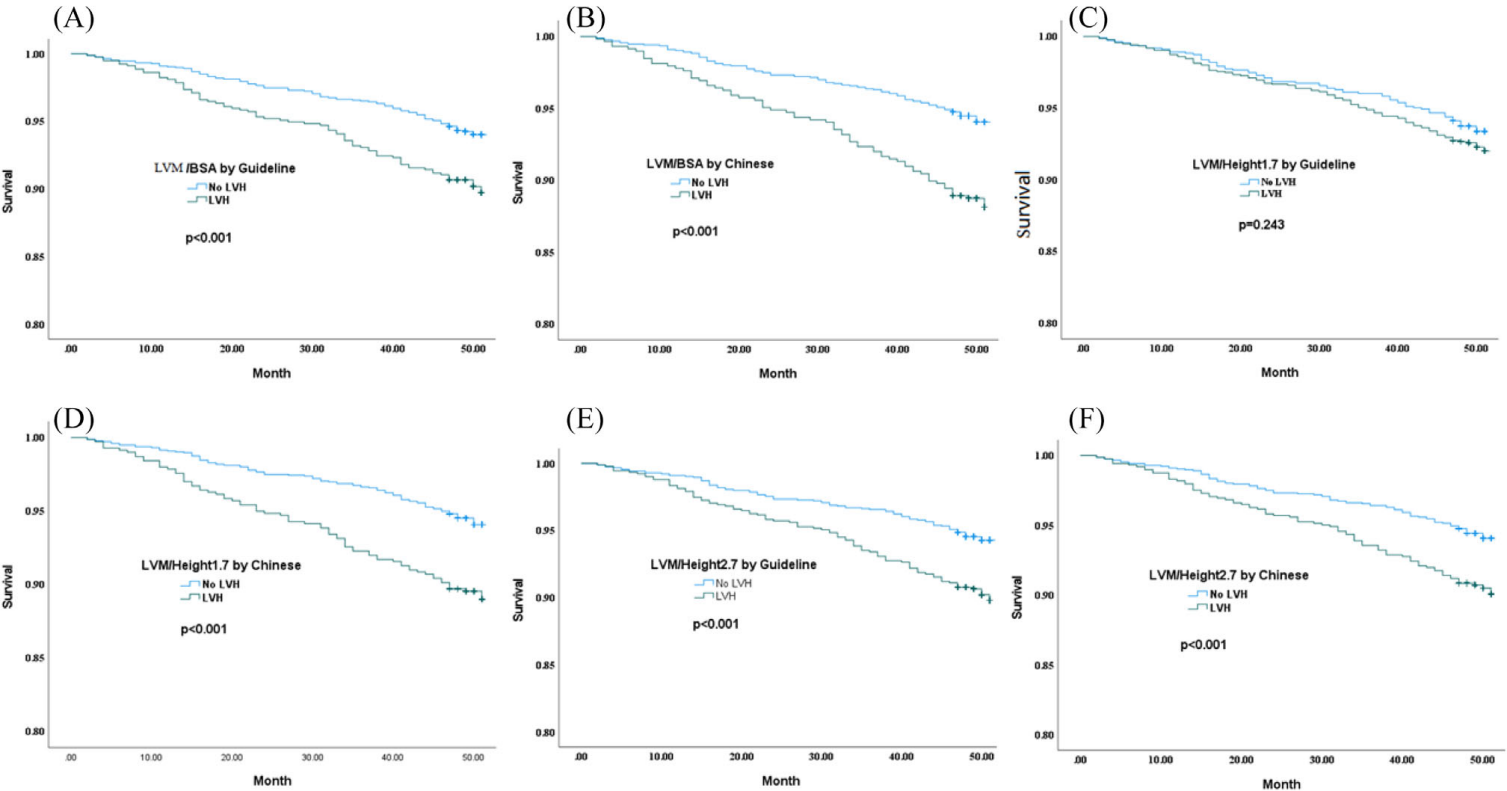
Kaplan‐Meier survival estimates of LVH and all‐cause mortality by different thresholds, LVH, left ventricular hypertrophy. (A) LVH defined by LVM/BSA by Guideline. (B) LVH defined by LVM/BSA by Chinese. (C) LVH defined by LVM/height^1.7^ by Guideline. (D) LVH defined by LVM/height^1.7^ by Chinese. (E) LVH defined by LVM/height^1.7^ by Guideline. F: LVH defined by LVM/height^1.7^ by Chinese.

Kaplan‐Meier curve showed that hypertensive patients with LVH, which using the LVM/BSA, LVM/Height^2.7^ and LVM/Height^1.7^, defined by the international guideline and the Chinese thresholds had higher mortality risk, except LVM/Height^1.7^ defined by international guideline (Figure 3A,B,D,E,F; Log‐rank ratio, all *p*<.05). After adjusting for covariates in Table 3, LVH, which using the LVM/BSA, defined by the Chinese thresholds was associated with all‐cause mortality (HR: 1.56, 95%CI: 1.14‐2.14), while LVH defined by the international guide was associated with all‐cause mortality with slightly lower hazard ratio (HR: 1.52, 95%CI: 1.08‐2.15). When indexing LVM to height^1^ ^.^ ^7^, LVH defined by the Chinese thresholds was associated with increased risk of all‐cause mortality (HR: 1.60, 95%CI: 1.17‐2.20) and almost similar hazard ratio 1.54(1.04‐2.27) by the international guideline. When indexing LVM to height^2^ ^.^ ^7^, LVH defined by Chinese threshold was not independently with all‐cause mortality (HR: 1.28, 95%CI: 0.91‐1.80), LVH defined by the international guideline was also not significantly associated increased risk of all‐cause mortality (HR: 1.28, 95%CI: 0.99‐1.93). LVH defined by LVM/height^1.7^ using Chinese threshold had the lowest AIC.

**TABLE 3:** Comparison of the effect of LVH defined by Chinese thresholds or Guideline thresholds on all‐cause mortality.

	Model 1	Model 2		Model 1	Model 2	
	Chinese thresholds* HR (95% CI)	Chinese thresholds HR (95% CI)	AIC	Guideline thresholds HR (95% CI)	Guideline thresholds HR (95% CI)	AIC
LVH						
LVM/BSA^a^	2.06 (1.52‐2.80)	**1.56 (1.14‐2.14)**	2437.71	1.70 (1.26‐2.30)	**1.52 (1.08‐2.15)**	2435.63
LVM/Height^1.7^ ^b^	1.93 (1.43‐2.62)	**1.60 (1.17‐2.20)**	2434.49	1.29 (0.95‐1.71)	**1.54 (1.04‐2.27)**	2438.47
LVM/Height^2.7^ ^c^	1.71 (1.27‐2.31)	1.28 (0.91‐1.80)	2438.63	1.80 (1.33‐2.43)	1.38 (0.99‐1.93)	2437.86

*Note*: Model 1: unadjusted

Model 2: adjusted for age, sex, current smoking, waist circumference, body mass index, systolic blood pressure, heart rate, total cholesterol, estimated glomerular filtration rate, type 2 diabetes mellitus, chronic kidney disease, coronary heart disease, stroke, anti‐hypertension drugs.

LVM: left ventricular mass; LVH, left ventricular hypertrophy; HR, hazard ratio; AIC: Akaike Information Criterion.

* Thresholds defined left ventricular and atrial remodeling in hypertensive patients using thresholds from international guidelines and EMINCA data.^10^

^a^ Guideline thresholds from recommendations for cardiac chamber quantification by echocardiography in adults: an update from the ASE and EACVI.^16^

^b^ Left ventricular mass: allometric scaling, normative values, effect of obesity, and prognostic performance.^19^

^c^ 2018 ESC/ESH Guidelines for the management of arterial hypertension.^18^

### 3.5 Predictive value of those LVH indicators for mortality

The predictive value of those LVH indicators was showed in Table 4. There was independent predictive value for most indicators except LVM/Height^2.7^ and LVM/Height^1.7^ defined by Guideline thresholds. Among those indicators, LVH defined by LVM/BSA using Chinese threshold was better associated with all‐cause mortality (C‐statistics 0.578) and cardiovascular mortality (C‐statistics 0.587). LVH defined by LVM/Height^1.7^ using Chinese thresholds was also better associated with all‐cause mortality (C‐statistics 0.576) and cardiovascular mortality (C statistics 0.578). In Table 5, the incremental value of LVH indicators for predicting mortality was showed. Only LVM/Height^1.7^ defined by Chinese thresholds had significantly increased the C‐statistics in the time of three‐year follow‐up. Although compared with the clinical model, adding LVH indicators slightly increased C‐statistics. For prediction of all‐cause mortality, LVM/Height^1.7^ by Chinese threshold had highest C‐statistics in all follow‐up time. For prediction of cardiovascular mortality, LVM/Heigh^1.7^ by Chinese threshold and LVM/BSA by Guideline had higher C‐statistics in all follow‐up time.

**TABLE 4:** The predictive value of single LVH indicators for mortality by ROC.

	All‐cause mortality		VS Guideline	Cardiovascular mortality		VS Guideline
	C‐statistics	*p*‐value	*p*‐value	C‐statistics	*p*‐value	*p*‐value
LVM/BSA by Guideline^a^	0.564	.005		0.573	.036	
LVM/BSA by Chinese	0.578	.001	.335	0.587	.012	.558
LVM/Height^1^ ^.^ ^7^ by Guideline^b^	0.523	.317		0.529	.408	
LVM/Height^1^ ^.^ ^7^ by Chinese	0.576	.001	<.001	0.578	.025	.031
LVM/Height^2^ ^.^ ^7^ by Guideline^c^	0.572	.001		0.563	.071	
LVM/Height^2^ ^.^ ^7^ by Chinese	0.566	.004	.339	0.568	.051	.606

Abbreviations: LVM: left ventricular mass; LVH, left ventricular hypertrophy. ROC: receiver operating characteristic curve. BSA: body surface area.

^a^ Guideline threshold from recommendations for cardiac chamber quantification by echocardiography in adults: an update from the ASE and EACVI.^16^.

^b^ Left ventricular mass: allometric scaling, normative values, effect of obesity, and prognostic performance.^19^

^c^ 2018 ESC/ESH Guidelines for the management of arterial hypertension.^18^

**TABLE 5:** The incremental value of LVH indicators for predicting mortality by time‐dependent ROC.

		Guideline	Chinese	Guideline	Chinese	Guideline	Chinese
All‐cause mortality	Model 1 clinical	Model 2 clinical + LVM/BSA^a^	Model 3 clinical + LVM/BSA	Model 4 Clinical+ LVM/Height^2.7^ ^c^	Model 5 clinical + LVM/Height^2.7^	Model 6 clinical + LVM/Height^1.7^ ^b^	Model 7 clinical + LVM/Height^1.7^
Month	Ref						
12	0.785	0.787	0.788	0.787	0.786	0.786	0.792
24	0.813	0.816	0.815	0.814	0.814	0.814	0.817
36	0.826	0.830	0.829	0.828	0.827	0.827	**0.831** *
48	0.818	0.820	0.820	0.820	0.819	0.819	0.821
50	0.789	0.791	0.791	0.791	0.790	0.790	0.793
Cardiovascular mortality							
Month	Ref						
12	0.825	0.832	0.831	0.826	0.826	0.822	0.833
24	0.853	0.863	0.860	0.855	0.855	0.854	0.862
36	0.827	0.835	0.833	0.828	0.829	0.828	0.836*
48	0.815	0.820	0.819	0.816	0.817	0.816	0.819
50	0.770	0.775	0.774	0.772	0.772	0.771	0.775

*Note*: Data showed C‐statistics.

Model 1: clinical model contains age, sex, current smoking, waist circumference, body mass index, systolic blood pressure, heart rate, total cholesterol, estimated glomerular filtration rate, type 2 diabetes mellitus, chronic kidney disease, coronary heart disease, stroke, anti‐hypertension drugs.

LVM: left ventricular mass; LVH, left ventricular hypertrophy. ROC: Receiver operating characteristic curve. Model 1 was the reference.

^a^ Guideline threshold from recommendations for cardiac chamber quantification by echocardiography in adults: an update from the ASE and EACVI^16^.

^b^ Left ventricular mass: allometric scaling, normative values, effect of obesity, and prognostic performance^19^.

^c^ 2018 ESC/ESH Guidelines for the management of arterial hypertension^18^.

* *p* < 0.05

### 3.6 Reproducibility

The intraclass correlation coefficients of interobserver reproducibility for IVSTd, LVEDD, LVPWTd were 0.94, 0.97, and 0.93, respectively (*p* < .001 for all). The intraclass correlation coefficients of intra‐observer reproducibility for IVSTd, LVEDD, LVPWTd were 0.96, 0.98, and 0.94, respectively (*p* < .001 for all). The Bland–Altman plots also demonstrated good agreement between interobserver reproducibility and intra‐observer reproducibility (Figure S1).

## 4 DISCUSSION

We revealed that LVH defined by the Chinese thresholds was more specific to identify LVH‐related mortality. LVM/BSA and LVM/Height^1.7^ defined by Chinese threshold had better predictive value for mortality and LVM/Height^1.7^ defined by Chinese threshold had significantly incremental value for mortality.

Recently, several studies have demonstrated that normative 2D echocardiographic parameters reference ranges differed significantly among different racial and ethnic groups,^8^, ^20^, ^21^, ^22^ including the EMINCA study,^9^ which proposed healthy Chinese thresholds. Recently, Sheng YY et al revealed that the current ASE/EACVI guideline thresholds might lead to significant misdiagnosis of abnormal LV geometry in Chinese patients with hypertension and proposed the Chinese thresholds for diagnosis of LVH.^10^ Previous study^8^ has demonstrated that the LVM of American women was larger than that of Chinese women. As we all know, the height of American women is higher than that of Chinese women. When indexing LVM to BSA or H^1.7^, lower height in Chinese women contributed to higher LVMI and threshold value for LVH. While indexing LVM to H^2.7^, the threshold values were nearly the same. When indexing LVM to BSA, LVH defined by Chinese thresholds was significantly lower in the present study. When indexing LVM to H^1.7^, LVH defined by Chinese threshold was lower than by Guideline threshold. When indexing LVM to H^2.7^, LVH proportion showed no significant difference.

Significant racial/ethnic differences in the relationship between LVM index (or LVH) and incident CVD has been demonstrated by Multi‐Ethnic Study of Atherosclerosis (MESA) involving 4 racial/ethnic groups (non‐Hispanic Whites, Chinese, Blacks, and Hispanics). LVH has more prognostic utility predicting future CVD events for Chinese (HR: 5.3, 95% CI: 1.6–17).^23^ Hence, compared with other races, LVH defined by Chinese threshold may have better utility when included in an ethnic‐specific risk‐stratification algorithm for Chinese. The similar relationship of racial/ethnic differences between electrocardiographic LVH and CVD mortality has been demonstrated.^24^ LVH contributed more CVD mortality in African Americans than it did in Whites. However, the study did not include Chinese population. Our studies showed that despite higher proportion of LVH defined by the international guideline thresholds, in contrast to LVH defined by the Chinese thresholds, LVH failed to predict cardiovascular mortality. The Chinese thresholds seem to better identify patients’ mortality risk by determine the worst cases. This issue is not specific to the Chinese subject but to patients of all races that undergo cardiac evaluations, like the studies mentioned above. Our study emphasizes the need for detailed interpretation of studies evaluating the predictive ability of LVH by racial/ethnic threshold.

Our results showed that the Chinese thresholds indexing BSA and height^1^ ^.^ ^7^ had the better predictive value for mortality than other body shape indicators and LVM indexing height^1^ ^.^ ^7^ by Chinese threshold had better incremental value for mortality. Previous study has suggested that LVM/Height^1.7^ was more specific than LVM/BSA or LVM/Height^2.7^ to identify obesity‐related LVH and consistently associated with cardiovascular events and all‐cause mortality.^19^ Normalization must exclude the impact of sex on LVM and body size. Currently, the common exponent was applied in both sexes, therefore allometric normalization itself cannot be influenced by the body size. Chinese are generally moderate in body type, with fewer obese people and lower height than Europeans and Americans. The reason for identifying an exponent which is not affected by sex itself is men are associated with higher cardiovascular events. LVH defined by LVM/height^1.7^ using Chinese threshold was consistently associated with all‐cause mortality, however, failed to predict cardiovascular mortality. Previous study showed normalization for height^1^ ^.^ ^7^ fully eliminates the ethnic‐independent, sex‐independent relationship between LVM and height.^19^ The incremental value of LVM/Height^1.7^ beyond clinical model also been revealed with using Chinese thresholds, not Guideline thresholds.

There are some limitations of our study. First, the Chinese threshold from EMINCA study was limited by the fact that it was obtained by age‐sex matching hypertensive patients. This threshold may not be generalizable to an otherwise general population. Second, we collected complete data on mortality but no other cardiovascular events. There were less cardiovascular mortality and less power to detect statistical significance. Although the number for cardiovascular mortality was small, LVM/BSA defined by Chinese thresholds have shown independent prognostic value for cardiovascular mortality, not guideline thresholds. Third, no probing biomarkers explain the potential underlying mechanisms involved in the relationship between LVH and mortality. Fourth, currently, our study did not have sufficient power to explore sex differences and further follow‐up was needed.

In conclusion, in community hypertensive populations, current international guideline thresholds may lead to significant overestimate of LVH and the mortality risk attributable to LVH that is identified with the Chinese thresholds is different from the international guideline thresholds. The potential value of this paper is that race‐specific thresholds should be used to classify LV hypertrophy related to mortality risk stratification. The ability to properly account for ethnic body size difference will allow for more accurate quantification of LVM in Chinese threshold.

## AUTHOR CONTRIBUTIONS

Dan Zhou, Anping Cai contributed to the conception and design of the study. Dan Zhou, Mengqi Yan drafted the manuscript. Dan Zhou, Mengqi Yan revised the manuscript. Mengqi Yan, Qiu Xie, Qi Cheng, Songtao Tang contributed to the acquisition of data, interpretation of data, and analysis of data. Dan Zhou, Anping Cai, Yingqing Feng contributed to the interpretation of data and critical revision of the article for important intellectual content. All authors contributed to the article and approved the submitted version.

## CONFLICT OF INTEREST STATEMENT

The authors declare no conflicts of interest.

## Supporting information

Supp Information

## Data Availability

The data used in the current study are available upon reasonable request to the corresponding author.
