# Supplementary material for: Prognostic implications of left ventricular hypertrophy defined by the thresholds from the international and Chinese guidelines
Source: J Clin Hypertens (Greenwich). 2023 Jun 19;25(7):628–37. doi: 10.1111/jch.14687 (PMC10339371; doi:10.1111/jch.14687)
Supplement: Supplementary file 1 — Supp Information [file JCH-25-628-s001.docx]

**Table S1 LVH defined by indexing LVM to BSA, height, height ^1.7^ and height^2.7^ respectively using different thresholds.**

|  | **Chinese thresholds*** | **Guideline thresholds** |
| --- | --- | --- |
| **LVM/BSA^a^** | >109g/BSA (male)  >105g/BSA (female) | >115g/BSA (male)  >95g/BSA (female) |
| **LVM/height^1.7b^** | >81g/H^1.7^(male)  >78g/H^1.7^(female) | >81g/H^1.7^(male)  >60g/H^1.7^(female) |
| **LVM/height^2.7c^** | >49g/H^2.7^ (male)  >48 g/H^2.7^ (female) | >50g/H^2.7^(male)  >47g/H^2.7^(female) |

LVH: left ventricular hypertrophy; LVM: left ventricular mass; BSA: body surface area.

* Thresholds defined left ventricular and atrial remodeling in hypertensive patients using thresholds from international guidelines and EMINCA data^10^.

^a^ Guideline thresholds from recommendations for cardiac chamber quantification by echocardiography in adults: an update from the ASE and EACVI^16^

b Left ventricular mass: allometric scaling, normative values, effect of obesity, and prognostic performance^19^

c 2018 ESC/ESH Guidelines for the management of arterial hypertension^18^

^
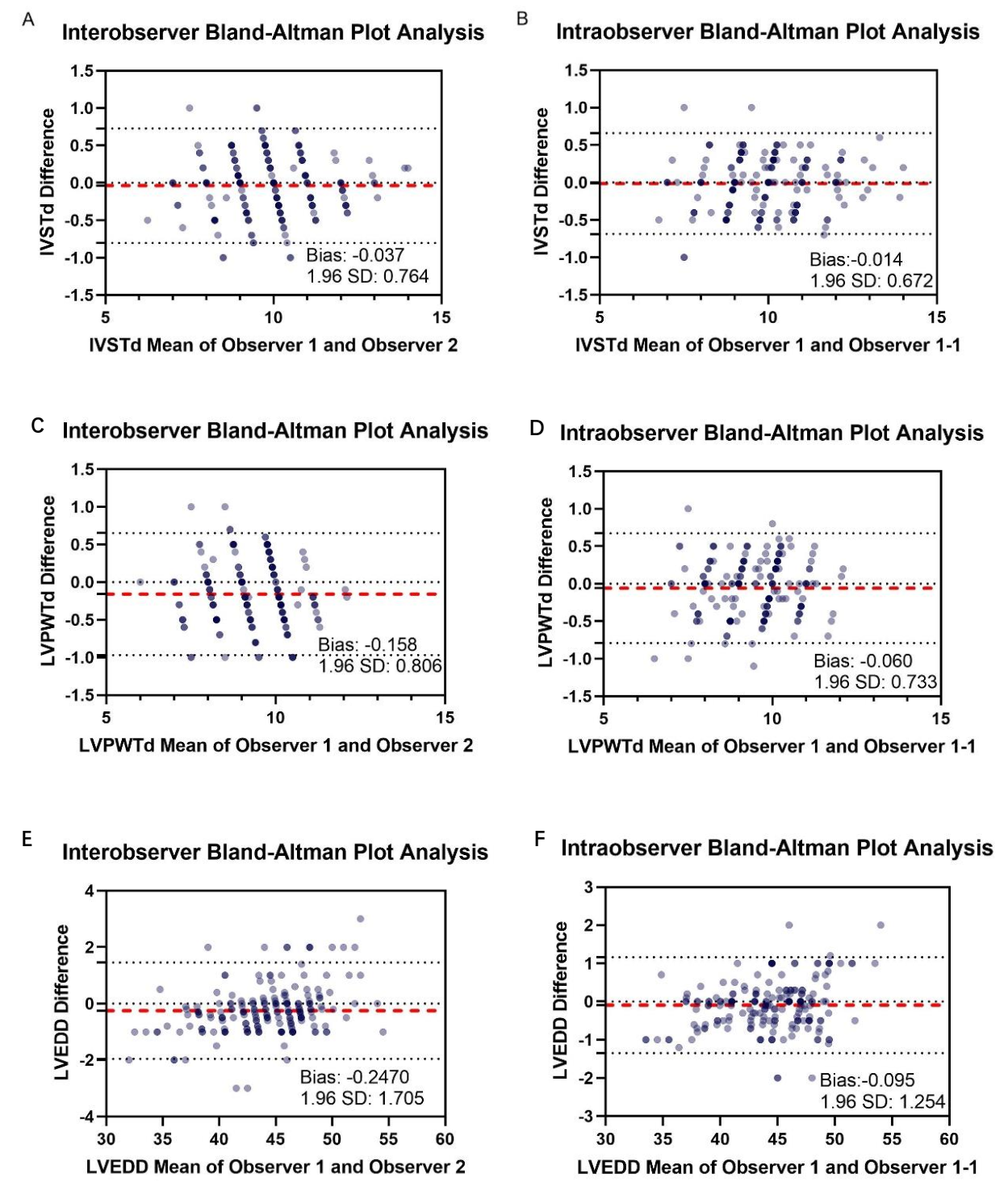
^

Figure S1. Bland-Altman plots of intraobserver and interobserver variability of echocardiographic parameters. Panel A and B: intraobserver and interobserver variability for interventricular septum thickness at end-diastole (IVSTd); Panel C and D: intraobserver and interobserver variability for left ventricular posterior wall thickness at end-diastole (LVPWTd); Panel E and F: intraobserver and interobserver variability for left ventricular end-diastolic diameter (LVEDD).


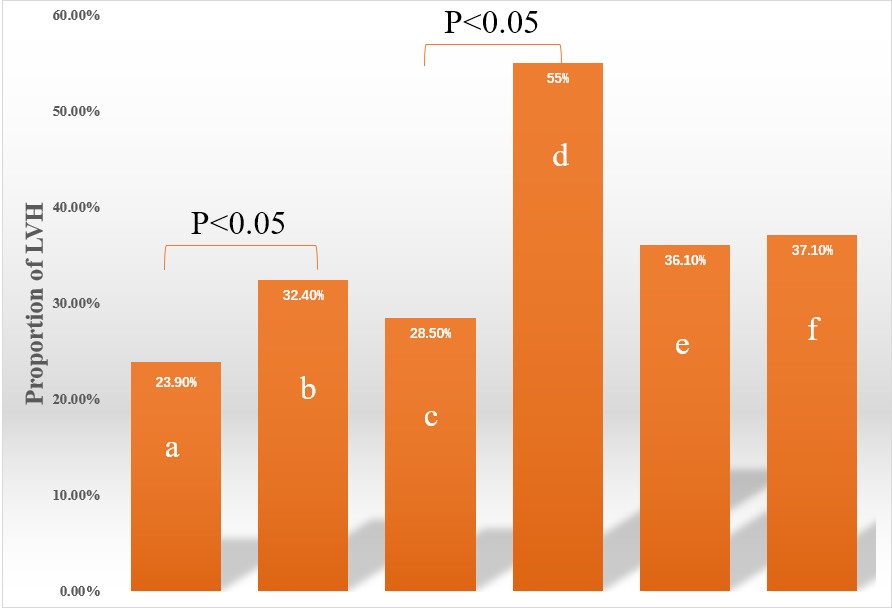


Figure S2 Prevalence of Left ventricular hypertrophy (LVH) in hypertensive patients using Chinese thresholds/BSA(a), Guideline thresholds/BSA(b), Chinese thresholds/H^1.7^(c), Guideline thresholds/H^1.7^(d), Chinese thresholds/H^2.7^(e) and Guideline thresholds/H^2.7^(f).
